# Report of clinico-pathological features of breast cancer in HIV-infected and uninfected women in Botswana

**DOI:** 10.1186/s13027-019-0245-6

**Published:** 2019-10-21

**Authors:** Rohini K. Bhatia, Mohan Narasimhamurthy, Yehoda M. Martei, Pooja Prabhakar, Jeré Hutson, Sebathu Chiyapo, Ignetious Makozhombwe, Michael Feldman, Mukendi K. A. Kayembe, Kum Cooper, Surbhi Grover

**Affiliations:** 1 University of Rochester School of Medicine and Dentistry, Rochester, NY USA; 2 Department of Pathology, Faculty of Medicine, University of Botswana, Gaborone, Botswana; 3 Division of Hematology – Oncology, University of Pennsylvania, Philadelphia, PA USA; 4 University of Texas Southwestern Medical Center, TX, Dallas, USA; 5 University of Pennsylvania, Philadelphia, PA USA; 6 Gaborone Private Hospital, Gaborone, Botswana; 7 Diagnofirm Medical Laboratories, Gaborone, Botswana; 8 Department of Pathology, University of Pennsylvania, Philadelphia, PA USA; 9 National Health Laboratory, Gaborone, Botswana; 10 Department of Radiation Oncology, University of Pennsylvania, 3400 Civic Center Blvd, Philadelphia, PA 19104 USA; 11 Princess Marina Hospital, Gaborone, Botswana; 12 Botswana University of Pennsylvania Partnership, Gaborone, Botswana

**Keywords:** Breast cancer, Sub-Saharan Africa, Breast cancer subtypes, HIV

## Abstract

**Background:**

To characterize the clinico-pathological features including estrogen receptor (ER), progesterone receptor (PR) and Her-2/neu (HER2) expression in breast cancers in Botswana, and to compare them by HIV status.

**Methods:**

This was a retrospective study using data from the National Health Laboratory and Diagnofirm Medical Laboratory in Gaborone from January 1, 2011 to December 31, 2015. Clinico-pathological details of patients were abstracted from electronic medical records.

**Results:**

A total of 384 unique breast cancer reports met our inclusion criteria. Of the patients with known HIV status, 42.7% (50/117) were HIV-infected. Median age at the time of breast cancer diagnosis was 54 years (IQR 44–66 years). HIV-infected individuals were more likely to be diagnosed before age 50 years compared to HIV-uninfected individuals (68.2% vs 23.8%, *p* < 0.001). The majority of patients (68.6%, 35/51) presented with stage III at diagnosis. Stage IV disease was not presented because of the lack of data in pathology records surveyed, and additionally these patients may not present to clinic if the disease is advanced. Overall, 68.9% (151/219) of tumors were ER+ or PR+ and 16.0% (35/219) were HER2+. ER+ or PR+ or both, and HER2- was the most prevalent profile (62.6%, 132/211), followed by triple negative (ER−/PR−/HER2-, 21.3%, 45/211), ER+ or PR+ or both, and HER2+, (9.0%, 19/211) and ER−/PR−/HER2+ (7.1%, 15/211). There was no significant difference in receptor status noted between HIV-infected and HIV-uninfected individuals.

**Conclusions:**

Majority of breast cancer patients in Botswana present with advanced disease (stage III) at diagnosis and hormone receptor positive disease. HIV-infected breast cancer patients tended to present at a younger age compared to HIV-uninfected patients. HIV status does not appear to be associated with the distribution of receptor status in breast cancers in Botswana.

## Background

Breast cancer is the most commonly diagnosed cancer among women worldwide. Mortality due to breast cancer disproportionately affects women in low- and middle-income countries (LMIC) [1]. Previous research has suggested that breast cancer in sub-Saharan Africa (SSA) tends to be more aggressive and present with unfavorable clinical and pathologic characteristics, which include presentation at a younger age with advanced stage disease, high histologic grade, and higher proportion of triple negative breast cancers (TNBC) [2–7]. However a recent systematic review of breast cancer molecular profile across Africa shows significant heterogeneity across the continent [8].

Botswana is an upper-middle-income country in SSA with a population of 2.2 million, and the third highest prevalence of HIV in the world, 17.6% among the general population [9–12]. Breast cancer is the second most common malignant disease (after cervical cancer) diagnosed among women in Botswana, with an age adjusted incidence rate of 17.5 per 100,000 women [13, 14]. There are few retrospective analyses of breast cancer clinical and molecular profiles in SSA [15–19], and of breast cancer and HIV presentation in the developed world and Africa [20–31], but no published studies to our knowledge have reported the characteristics of breast cancer and HIV in Botswana. Such studies are needed to improve our understanding of breast cancer biology in Africa and also among HIV-infected patients to improve treatment outcomes.

The aim of this retrospective review was to characterize the clinico-pathological features, including age at diagnosis, stage at presentation, estrogen receptor (ER), progesterone receptor (PR), and Human Epidermal Growth Factor Receptor2 (Her-2/neu) of breast cancer pathology specimens reviewed at the National Health Laboratory (NHL) and Diagnofirm Medical Laboratory (DML) in Gaborone, Botswana.

## Methods

The NHL is located in Gaborone and currently serves as a referral laboratory for the southern region of the country providing services to 14 district hospitals, including Princess Marina Hospital, a tertiary referral center. DML is an independent laboratory in Botswana with twelve locations throughout the country. While NHL sees about 85% of the breast cancer patients in Botswana, DML sees about 15% [32]. Archival pathology reports are reported and stored in the respective electronic medical record systems - Integrated Patient Management System (IPMS) at NHL and Sukra Laboratory Information Management System (SLIMS) at DML.

We conducted a retrospective analysis using archival data from NHL and DML in Gaborone for breast cancer pathology specimens analyzed and reported from January 1, 2011 to December 31, 2015. The data includes all patients who underwent a biopsy, wide local excision and mastectomy for breast cancer. We manually abstracted available data from the pathology report: age, gender, tumor laterality, tumor grade, tumor size and nodal status; and demographic data and information on HIV status from the electronic medical record. Because information was ascertained from pathology records and breast and lymph node procedures only, data on metastatic sites were not available. Staging data was computed according to AJCC v7, using tumor size and lymph node status from breast surgical specimens with the assumption that patients who underwent definitive surgery had been ruled out for metastatic disease. AJCC v7 vs v8 was utilized as we used retrospective staging records and did not actively stage tumors. Stage IV patients were not presented in our data. Pathology records reported breast an axillary lymph node staging only, while most stage IV disease is diagnosed both radiographically and clinically and available in individual patient notes, we were unable to account for these in the pathology records.

### Immunohistochemistry

ER, PR, and HER2 immunostaining was performed in an automated stainer at NHL and manually at the DML at the time of diagnosis as part of routine care. All staining was done using a standardized protocol under optimum physical conditions as prescribed by the manufacturer. In brief, formalin fixed, paraffin-embedded breast tumor blocks were obtained and 4-mm tissues were sectioned, deparaffinized, and rehydrated. Antigen retrieval was performed by using a microwave in Tris/EDTA buffer, pH 9.0 (for anti - ER and PR) and Citrate buffer, pH 6.1 (for anti – HER2/neu) at 97 °C for 20 min and blocked with Phosphate buffer containing hydrogen peroxide, 15 mmol/L NaN3 and detergent (Dako EnVision™ FLEX Peroxidase-Blocking Reagent). Tissue sections were then incubated for 30 mins with primary antibodies at room temperature: anti-ER (clone EP1; DAKO), anti-PR (clone PgR636; DAKO), and anti-HER2/neu (Anti human c-erbB2 Oncoprotein; Dako), followed by goat secondary antibody molecules against rabbit and mouse immunoglobulins (EnVision™ FLEX /HRP, Dako). Staining was visualized by using diaminobenzidine (EnVision™ FLEX DAB+ Chromogen; Dako) and counterstained with hematoxylin. IHC manufacturers and sources are Dako Products, Agilent Technologies, USA.

Immunohistochemical stains were evaluated using standardized controls provided by the manufacturer. ER, PR and HER 2/neu status were assessed semi-quantitatively using Standard Allred scoring system. ER and PR were considered positive if > 1% nuclei of tumor cells stained [21]. HER2 was scored as 0, 1+, 2+, or 3+. Fluorescent in situ hybridization (FISH) was not performed for intermediate 2+ HER2 due to inability for in-country FISH analysis. For analytical purposes, only a score of 3+ was considered HER2 +, whereas scores ≤2+ were assumed to be HER2 -.

### Statistical analysis

Data from all pathology reports were extracted and recorded in a Research Electronic Data Capture (RedCap) database. Descriptive statistics was used to quantify mean, median, range and respective proportions. We also performed a chi squared test to compare the equality of proportions of age > 50 (to stratify based upon pre and post-menopausal), receptor status, and grade at initial breast cancer diagnosis between HIV-infected and HIV-uninfected breast cancer patients. All analyses were performed in STATA.

### Ethical review

This study was reviewed and approved by the Institutional Review Board of the University of Botswana, Health Research and Development Committee of Ministry of Health and Wellness and the Institutional Review Board of DML.

## Results

### Patient population

There were 409 breast pathology reports analyzed from January 2011 to December 2015. Seventeen were excluded because biopsies were taken before 2011 or because they were not breast/axillary lymph node biopsies, and 8 were excluded because of insufficient residual tumor for analysis. Using one sample per patient, a final total of 384 reports of pathologically confirmed breast cancer cases were included in our analysis. Six of these patients were male.

### Clinico-pathological features

#### Age

Age was reported on 330 (85.9%) reports (Table 1). Median age at the time of breast cancer diagnosis was 54 years (range 27–96 years). Forty percent (134/330) of reports were from patients who were less than 50 years of age and 18.8% (62/330) of reports were from patients less than 40 years of age.

**Table 1:** Demographic characteristics and distribution of staging of breast cancer diagnosed in Botswana from 2011 to 2015

Age	n = 330
< 50	134 (40.6%)
≥50	196 (59.4%)
Gender	n = 384
Female	378 (98.4%)
Male	6 (1.6%)
HIV Status	n = 114
HIV-infected	49 (42.9%)
HIV-uninfected	65 (57.02%)
CD4 Count	n = 42
< 250	13 (30.9%)
250–500	17 (40.5%)
> 500	12 (28.6%)
TNM Staging	n = 51
Early Stage	16 (31.4%)
Stage I A	2 (3.9%)
Stage I B	0 (0.0%)
Stage II A	9 (17.7%)
Stage II B	5 (9.8%)
Advanced Stage	35 (68.6%)
Stage III A	23 (45.1%)
Stage III B	10 (19.6%)
Stage III C	2 (3.9%)

#### Laterality

Left breast was involved in 42.0% (161/384) of reports and right breast in 40.0% (153/384) of reports. Bilateral breast cancers were reported in 3 cases. There was no mention about the laterality in 17.4% (67/384) cases. There was no predilection for the laterality as no significant difference was noted between the proportions of left vs. right breast cancers.

#### Tumor type and stage

The most common histologic subtype was invasive ductal carcinoma (78.12%, 300/384). Second most common type was mucinous carcinoma (6.25%, 24.6/384) followed by medullary (4.6%, 18/384) and invasive papillary (2.34%, 9/384). Invasive lobular carcinoma constituted 1.53% (6/384) of all breast cancers. Presence of ductal carcinoma in-situ (DCIS) was not specified in 90% (344/384) cases, but in those where it was specified, DCIS was present in 90% (36/40) of cases along with invasive carcinoma. Of the six male breast cancers, four were invasive ductal carcinomas.

Complete tumor and nodal staging was reported on 51/384 specimens (13.3%). Mean tumor size was 4.3 cm (range 0.8–16 cm). Four percent of patients presented with Stage I disease (*n* = 2), 27.4% presented with Stage II (*n* = 14), and 68.6% (*n* = 35) presented with stage III breast cancer (Table 1).

#### Histologic grade

Over half (53.2%, 157/295) of the tumors were of histologic grade 2, with a quarter (25.8%, 76/295) presenting with features of grade 3. Eighty-nine reports either had grade noted as “not specified” or were missing grade information. Ki-67 status was available for only 12/211 tumor samples. This is a common data challenge for pathology samples in Botswana.

#### Receptor status

Immunohistochemistry was completed on 219 of the samples at the time of their removal. From retrospective review, eight of the samples had at least one receptor type noted as “not specified,” allowing for complete IHC profile to be completed on 211 samples. Overall, 69.2% (148/214) of tumors were ER+, 52.3% (112/213) of tumors were PR+, and 16.4% (35/214) were HER2+ overexpressed. 62.6% of samples included a profile of ER+ or PR+ or both, and HER2- (ER/PR+/HER2-, 132/211), 21.3% were triple negative (ER/PR/HER2-, 45/211), 9.0% were ER+ or PR+, or both, and HER2+ (ER/PR/HER2+, 19/211) and 7.1% were ER−/PR−/HER2+ (15/211). ER−/PR−/HER2+ cancers were more likely to be seen in patients ≥50 years of age (58.3% vs. 41.7%) (Table 2). Of the five males for whom immunohistochemistry was completed, three had ER+ tumors. Only 28 samples had both receptor status and staging information available.

**Table 2:** Immunohistochemistry based molecular subtypes and pathological features for breast cancers diagnosed in Botswana between 2011 and 2015

	*ER/PR+/HER2- n (%)	ER/PR+/HER2+ n (%)	ER−/PR−/HER2 + HER2 enriched n (%)	ER−/PR−/HER2-Triple Negative n (%)
	132 (62.6%)	19 (9.0%)	15 (7.1%)	45 (21.3%)
Histologic grade				
Grade 1	33 (25.0)	5 (26.3)	1 (6.7)	2 (4.4)
Grade 2	59 (44.7)	10 (52.6)	8 (53.3)	23 (51.1)
Grade 3	21 (15.9)	3 (15.8)	3 (20.0)	13 (28.8)
Not specified/missing	19 (14.4)	1 (5.3)	3 (20.0)	7 (15.5)
Age				
< 50 years	50 (37.8)	10 (52.6)	5 (33.3)	21 (46.7)
≥ 50 years	64 (48.5)	6 (31.6)	7 (46.7)	19 (42.2)
Age not specified/missing	18 (13.6)	3 (15.8)	3 (20.0)	5 (11.1)
Stage				
Stage I and Stage II	5 (29.4)	0 (0.0)	0 (0.0)	2 (28.6)
Stage III	12 (70.6)	1 (100.0)	3 (100.0)	5 (71.4)

*ER/PR+/HER2-: ER+ or PR+ or both, and HER2-

ER/PR+/HER2+: ER or PR+ or both, and HER2+

ER−/PR−/HER2+: ER – and PR- and HER2+

ER−/PR−/HER2-: ER- ad PR- and HER2-

#### HIV status

Twenty-nine percent (114/384) of patients had known HIV status; of those patients, 43.0% (49/114) were HIV-infected and 57.0% (65/114) were HIV-uninfected. The median CD4 count among HIV-infected patients with breast cancer was 396 cells/mm^3^ (IQR 209 cells/mm^3^–525.6 cells/mm^3^). Antiretroviral treatment (ARV) coverage was not available for this population, however ARV coverage in Botswana is well-documented to be 84% among adults [33]. Table 3 shows stratification of age, receptor status, and histologic grade of tumor by HIV status. HIV-infected patients with breast cancer were more likely to be younger (< 50 years of age) compared to HIV-uninfected patients (*p* < 0.0001). HIV-uninfected individuals had a slightly higher prevalence of ER+ disease than HIV-infected individuals (82.9% vs 70.0%), but this was not statistically significant. This trend was not seen for HER2+ and TNBC; however, our sample size for these groups were small (*n* = 16 and *n* = 9, respectively (Table 3).

**Table 3:** Distribution of receptor status, age and tumor grade for breast cancers diagnosed in Botswana between 2011 and 2015 by HIV status

Clinicopathological features	HIV-uninfected (*n* = 65) n (%)	HIV-infected (*n* = 49) n (%)	*p* value
Age			*p < 0.0001*
< 50	15 (23.0)	30 (61.2)	
≥ 50	48 (73.8)	14 (28.6)	
Unspecified	2 (3.1)	5 (10.2)	
Receptor Status			*p = 0.513*
ER+ or PR+ or both, HER2-	28 (43.1)	24 (48.9)	
ER+ or PR+ or both, HER2+	6 (9.2)	5 (10.2)	
HER2 enriched	1 (1.5)	4 (8.2)	
Triple Negative	4 (6.5)	5 (10.2)	
Missing	26 (40.0)	11 (22.4)	
Histologic Grade			*p = 0.481*
Grade 1	9 (13.8)	11 (22.4)	
Grade 2	25 (38.4)	24 (48.9)	
Grade 3	16 (24.6)	8 (16.3)	
Missing/Unspecified	15 (23.0)	6 (12.2)	

## Discussion

To our knowledge, this study analysing data on 384 patients with breast cancer is the first large pathology-based study describing clinical and pathological features of breast cancer in Botswana. We noted that breast cancer in Botswana was largely hormone receptor positive and tended to present more frequently at stage III than stages I and II. Median age at diagnosis for our cohort was 54 years, seven years younger than the median age (61 years) of both African-American and Caucasian women in the United States (U.S.), with 40% of women diagnosed before 50 years of age. Comparatively, median age of breast cancer diagnosis in the U.S. is 58 years for African-American women and 62 years for white women [22]. A previous review found that the median age of diagnosis in SSA is lower than in higher income countries, regardless of HIV status [19]. Hypotheses for this younger age include population structure of the region coupled with higher fertility, shorter life expectancy, and lower risk factors in older generations for breast cancer as opposed to biological differences in disease aggressiveness between Black African and Western Caucasian populations [23]. Recent data have found that Batswana have experienced a decline in total fertility rate (from 6.6 in 1981 to 2.8 in 2011) [34] and longer life expectancy (from 48.7 years in 2001 to 65.8 years in 2015) [35], suggesting that these may not be contributing factors to our study’s younger age at diagnosis. Though, breast cancer presentations in our analysis may not yet reflect the increase in life expectancy noted in 2015. While in the U.S., 28.1% of the population is > 55 years of age, in Botswana only 8.7% falls within that range, suggesting a natural population structure heavily dependent on a younger age. This further contributes to the idea that the younger age at breast cancer presentation in Botswana may be a result of population structure and not a biological tumor difference, especially in this context where majority of breast cancer cases present with a more favorable hormone receptor positive subtype [36, 37].

In our study, 67.6% of breast cancer cases were ER+, similar to data published in SSA and parts of East Africa, but different from West Africa where studies have reported a low rate of ER+ cases (35%) and high triple negative subtypes [8]. The prevalence of ER+ tumors among U.S.-born white females is 79%, while those of African-American females is lower, 61% [38]. Risk factors for breast cancer are known to be subtype specific. Differences in the incidence of receptor-specific breast cancer may be due to varying risk factors present in different ethnic groups. Although some experts in the field suggest that the high rates of triple negative breast cancers in West Africa may be due to poor pathology specimen handling and prolonged ischemic times, quality control studies and molecular profiling point to true molecular heterogeneity of breast cancer across sub Saharan African and even within specific countries [39]. Further studies and quality molecular pathology are needed to elucidate this further.

The prevalence of TNBC in Botswana (21.3%) was higher than those documented in white populations (10–16%) [40, 41] but lower than those seen in African-American (26%) [42]. While some studies have determined that premenopausal obesity and higher parity are correlated with aggressive TNBC receptor-type in the U.S. [43, 44], one Nigerian study looking at nearly 2000 breast cancer patients had failed to identify such a correlation [45]. In addition to low fertility rates, over half of Botswana’s population met the criteria for being overweight. Since obesity and low fertility both contribute to TNBC in the US, it is worth investigating further if the same factors that contribute to aggressive TNBC in African-American also contribute to it in Botswana, subsequently explaining relatively lower ER+ rates [46]. Finally, among receptor-status data, HER2+ status in Botswana was higher than those seen in US-born white and African-American women (16% vs 6–10%) [15, 25, 42]. In comparison to other SSA studies, HER 2+ status in Botswana was lower (16% vs 26.4%) [8].

Breast cancers in Africa are often characterized by a higher proportion of advanced disease, including large median tumor diameter and advanced stage. On average, 60% of black Africans in SSA present with advanced stage disease compared to 27% among white women and 32% among African-American women [23]. Seventy-two percent of our cohort presented with advanced stage disease. In our study, we defined advanced disease as at least stage III disease. As we were limited to pathology tissue samples, we did not have data on imaging that would confirm metastasis. Stage III breast cancer disease presentation is still considered “advanced” and this data is consistent with other data from SSA. In a large international study of breast tumor pathology, the African Breast Cancer-Disparities in Outcomes (ABC-DO) noted that of 1795 women, 1091 (61%) were diagnosed at advanced stage (III or IV) and 15% were stage IV with the lung and liver being the most common sties of metastasis [47].

This may be a reflection of delayed recognition of symptoms, diagnosis, or treatment or an indication of more aggressive tumor biology [48]. Delay from noticing a breast abnormality to time of presentation ranges from 2 weeks to 11 years across Africa [49]. In Botswana, the median delay from symptom onset to diagnosis, for all cancers, was 29 days [50]. Additionally, a recent review of turnaround time (TAT) of breast pathology samples found that the median delay from specimen collection to report sign out was 17 days for a biopsy specimen, and 39 days for a surgery specimen [51].There may be multiple contributing factors to advanced presentation, including genetic, biologic, and systematic delays, in the prevalence of advanced stage disease at diagnosis in Africa.

HIV prevalence among women in Botswana is 9.5%, and total HIV prevalence among adults aged > 15 years old is 24% [11, 52]. The prevalence of HIV in our Botswana study population of breast cancer patients (43.0%) was almost twice the national average, though only 30% of our study population had known HIV status perhaps in a biased population, potentially overestimating the impact of HIV in this population.

Major strengths of this study include documentation and characterization of over 200 breast cancer specimens in a five-year period in Botswana, where little data exists about the characteristics of breast cancer. Diagnostic information on the cancer was acquired from histopathological assessment as opposed to clinical chart review, assuring accuracy of diagnostic information. This data provides much-needed information for further studies looking to analyze the treatment and survival of breast cancer in Botswana, especially for younger and HIV-infected patients. It also establishes a baseline to understand the current practice of pathology and how it contributes to cancer care.

Limitations of this study include small sample size and retrospective nature of the study.

We do not assume a selection bias as both pathology centres serve the surrounding population. We are limited by the data found in medical records. Since staging data was pulled from pathological records, we do not have information on metastasis limiting our ability to characterize stage IV disease. There are challenges present in acquiring staging information, especially M stage with limited imaging resources and lack of trained staff to stage appropriately. Two retrospective reviews have evaluated completeness of breast cancer pathology reports across sub-Saharan Africa, both which identified substantial gaps documentation of basic pathology information [53, 54]. It is important to note that these reviews were done in West Africa and thus may not be applicable to Botswana and its resources. Nevertheless, a dearth of data exists on the state of pathology completeness in this region and its importance is underscored by this review. In this retrospective review, we were limited to the information from pathology reports, and limited by the challenges for physicians to fill out these reports. There are currently steps being taken to train staff in the recently initiated breast clinic to stage all patients prior to treatment and provide appropriate clinical information to pathology staff with the biopsy specimens. We need future prospective studies to get better staging information. Further, missing information on laterality was also present due to lack of availability in the electronic medical system. HIV data was only available for a third of our study population, and conclusions made from this data should not be overestimated. Both HIV status and tumour stage were available on a limited number of the patients due to missing information in medical records. Thus, no inferences could be made regarding the influence of HIV on stage at presentation. Fluorescent in situ hybridization was not performed for HER2 equivocal results because it is not available in the country, and thus HER2 was solely determined by IHC. This could underestimate HER2+ breast cancer and overestimate the cases that were HER2 negative. Moreover, laboratory procedures were performed in two different laboratories, possibly leading to technical differences in assessment of IHC status.

Despite limited staging info, our data provides important data on HIV, receptor status and age of breast cancer patients in Botswana and contributes to literature on this topic from SSA. This will help guide future studies on breast cancer patients with HIV as well as breast cancer screening and treatment strategies.

## Conclusion

Breast cancer patients in Botswana present at a younger age compared to their Western counterparts, but at a similar age compared to other SSA countries. Additionally, we found a higher prevalence of HIV among our population of breast cancer patients compared to the general Botswana population. HIV-infected individuals tended to present at a younger age at initial diagnosis compared to HIV uninfected individuals. Further investigations into the link between HIV and breast cancer, and understanding the impact on treatment are needed. Immunohistochemistry (IHC) of breast tumors seen is Botswana is similar to other countries in Southern and East Africa, with a fifth of cancers expressing triple negative type. There is no difference in receptor status by HIV-status. To our knowledge, this is the first attempt to characterize the clinico-pathological and immunohistochemical characteristics of breast cancer in Botswana, providing essential data to a growing body of knowledge of tumor biology in SSA.

## Data Availability

The dataset used and analyzed during the current study are available from the corresponding author on reasonable request.
